# Association of Physical Activity with Anthropometrics Variables and Health-Related Risks in Healthy Male Smokers

**DOI:** 10.3390/ijerph19126993

**Published:** 2022-06-07

**Authors:** Vijayamurugan Eswaramoorthi, Muhammad Zulhusni Suhaimi, Mohamad Razali Abdullah, Zulkefli Sanip, Anwar P. P. Abdul Majeed, Muhammad Zuhaili Suhaimi, Cain C. T. Clark, Rabiu Muazu Musa

**Affiliations:** 1 Faculty of Health Science, School of Rehabilitation Science, Universiti Sultan Zainal Abidin, Gong Badak Campus, Kuala Terengganu 21300, Terengganu, Malaysia; vijayeswar@unisza.edu.my; 2 Department of Allied Health Sciences, Faculty of Science, Universiti Tunku Abdul Rahman (UTAR), Bandar Barat, Kampar 31900, Perak, Malaysia; 3 East Coast Environmental Research Institute, Universiti Sultan Zainal Abidin, Kuala Terengganu 21300, Terengganu, Malaysia; razaliabdullah@unisza.edu.my; 4 Central Research Laboratory, School of Medical Sciences, Universiti Sains Malaysia, Kubang Kerian 16150, Kelantan, Malaysia; zulkefli@usm.my; 5 Innovative Manufacturing, Mechatronics and Sports Laboratory, Faculty of Manufacturing Engineering, Universiti Malaysia Pahang, Pekan 26600, Pahang, Malaysia; amajeed@ump.edu.my; 6 School of Robotics, XJTLU Entrepreneur College (Taicang), Xi’an Jiaotong-Liverpool University, Suzhou 215123, China; 7 Centre for Fundamental and Continuing Education, Universiti Malaysia Terengganu, Kuala Terengganu 21030, Terengganu, Malaysia; m.zuhaili@umt.edu.my; 8 Centre for Intelligent Healthcare, Coventry University, Coventry CV1 5FB, UK; ad0183@coventry.ac.uk

**Keywords:** anthropometrics variables, healthy smokers, health risks, physical activity, preventive healthcare, multivariate analysis

## Abstract

Anthropometric variables (AV) are shown to be essential in assessing health status and to serve as markers for evaluating health-related risks in different populations. Studying the impact of physical activity (PA) on AV and its relationship with smoking is a non-trivial task from a public health perspective. In this study, a total of 107 healthy male smokers (37 ± 9.42 years) were recruited from different states in Malaysia. Standard procedures of measurement of several anthropometric indexes were carried out, and the International Physical Activity Questionnaire (IPPQ) was used to ascertain the PA levels of the participants. A principal component analysis was employed to examine the AV associated with physical activity, k-means clustering was used to group the participants with respect to the PA levels, and discriminant analysis models were utilized to determine the differential variables between the groups. A logistic regression (LR) model was further employed to ascertain the efficacy of the discriminant models in classifying the two smoking groups. Six AV out of twelve were associated with smoking behaviour. Two groups were obtained from the k-means analysis, based on the IPPQ and termed partially physically active smokers (PPAS) or physically nonactive smokers (PNAS). The PNAS were found to be at high risk of contracting cardiovascular problems, as compared with the PPAS. The PPAS cluster was characterized by a desirable AV, as well as a lower level of nicotine compared with the PNAS cluster. The LR model revealed that certain AV are vital for maintaining good health, and a partially active lifestyle could be effective in mitigating the effect of tobacco on health in healthy male smokers.

## 1. Introduction

Tobacco addiction remains a significant global health problem, killing over 16 million people annually [1]. Owing to the rapid growth of the global population, the number of smokers is projected to rapidly increase. However, for the past four decades, relatively significant reductions in the prevalence of smoking have been witnessed globally. Nonetheless, challenges remain, especially in developing countries as well as countries with less stringent legislation. For instance, tobacco companies are directing their advertising to countries with less strict legislation. For example, tobacco reports and future trends in many African countries are showing that the development of smokers will remain high [2]. This phenomenon becomes a threat to the health of the world’s population that needs more intensive efforts to both control its usage and manage its effects on health.

Cigarette smoking is known to be directly implicated in several health-related problems. For instance, the ingestion of tobacco has been shown to increase the risks of hypotension [3,4]. On the other hand, lifestyles variables are shown to play a significant role in differentiating several categories of individuals, specifically, current smokers, ex-smokers, and non-smokers [5]. Indeed, it has been demonstrated, in a population-based cross-sectional study, that heavy smoking is associated with unhealthy lifestyles and overweight/obesity [6,7,8].

Smoking, coupled with a sedentary lifestyle (physical inactivity), is considered one of the major risk factors associated with non-communicable diseases. It is worth noting that not only do these factors foster the development of non-communicable diseases independently, but they also occur concurrently [9,10]. For instance, evidence has demonstrated an important negative association between physical exercise and risk for coronary heart disease such as angina, heart attack, heart failure, and abnormal heart rhythm [11], whilst a cohort analysis of US female nurses showed that PA, including moderate-intensity exercise such as walking, is correlated with a major reduction in the risk of stroke [12]. Similarly, more contemporary epidemiological studies have found that smoking can influence certain behavioural risk factors for chronic diseases, such as a lack of regular PA and poor dietary intake [13,14,15]. Moreover, recent empirical data have demonstrated an inverse correlation between smoking and PA in adults [16].

Research has highlighted that an inverse association between smoking and body weight is present, suggesting that an average smoker typically weighs less than a similar-aged non-smoker [17]. Hitherto, it was documented that significant variation exists between smokers and non-smokers in nutritional intake. Indeed, it was demonstrated that smokers are likely to consume diets that constitute a greater proportion of fats and a lesser amount of fruits and vegetables, as well as less dietary fibre [18,19]. Moreover, studies have consistently reported a higher intake of alcohol and caffeine among smokers relative to non- or ex-smokers [20,21].

Regarding energy intake, mixed findings have been reported. It was established that leptin, an adipocyte-derived signal molecule, interacts with some specific receptors located in the central neural system and peripheral tissues; these interactions often result in the decline of food intake and consequently trigger an increase in energy expenditure [22]. This suggests that nicotine concentration may elevate leptin levels in the body [23]. However, the resulting association between leptin concentration and smoking is still inconclusive, i.e., whether leptin concentration is triggered by smoking is yet to be determined [23,24,25]. For instance, smokers have been reported to consume similar or even higher energy compared with non-smokers regardless of body weight [19].

Malaysia has made advances in tobacco control in recent years; however, the prevalence of smoking among Malaysian people aged 15 years and above remains high despite the various anti-smoking measures implemented over recent decades. It has been reported that over 5 million Malaysian adults are smokers, which is equivalent to about 22.8 per cent of the entire population [26]. Moreover, the prevalence of smoking behaviour is highly skewed towards males, with approximately 43 per cent of males engaging in smoking behaviour, compared with 1.4 per cent of females, across all socio-demographic settings [27]. Clearly, these data suggest that more efforts are still needed to curb the prevalence of smoking for the well-being of the population.

AV are shown to be essential in assessing individuals’ health status and to serve as a marker for evaluating health-related risks in different populations [28,29]. Numerous investigations have been carried out to ascertain the anthropometric differences between smokers and non-smokers in different populations [30,31]. Some studies have focused on the nutritional status and physical activity level of the smoking group [13,14,32,33]; however, few or no studies have thus far focused on anthropometric variables and their relationships with PA among healthy adult smokers. Investigating the relationships between the aforementioned variables could be useful from a public health perspective in providing relevant information that could assist in converting pervasive smoking behaviour, as well as mitigating the long-term effects of smoking through exercising and other means.

Gender plays a significant role in the tendency to smoke in Malaysia, where the male-to-female ratio of smokers in the country is highly skewed: A larger proportion of smokers in the country are males, which could be attributed to the social norms, which are not favourable to female smoking [34]. Hence, the current study focused on adult male smokers only. To elucidate the relationship between AV and PA among healthy adult male smokers, the following research questions were devised to guide this study:

1. What are the most dominant AV characteristics of healthy male smokers?
2. What are the smokers’ PA levels and the most dominant AV characteristics that differentiate the PA groups?
3. How effective are the discriminant analysis (DA) and logistic regression models in discriminating and classifying the PPA and PNA smokers based on their AV characteristics?

## 2. Materials and Methods

### 2.1. Study Design

An ex post facto study design was used in the current study. This design was selected because the natural characteristics of the samples are not required to be manipulated or altered [35]. Essentially, the ex post facto design entails an investigation to find answers after an event occurred. Thus, the investigation was directed towards analysing the cause-and-effect relationships between the study variables.

### 2.2. Samples Size and Samples with the Inclusion and Exclusion Criteria

A preliminary power analysis using G*Power was carried out to ascertain the sample necessary to draw meaningful conclusions in the study [36]. A power computation of multivariate analysis with a power equivalent to 0.95 and alpha of 0.05 revealed that a sample size of 107 samples would be sufficient to detect a medium effect size of 0.25 (Cohen’s *F*), as suggested in a previous study [37]. Therefore, purposive sampling was used to recruit the participants. The participants in the current study had no history of any chronic diseases and were neither on routine medication nor alcohol consumers.

### 2.3. Data Collection

#### 2.3.1. Anthropometric and Health-Related Variables Measurement

The height of the participants was determined whilst the participants were in a standing position and unshod, using a portable stadiometer (206, Seca, Hamburg, Germany), while weight was assessed using a standard weighing scale. The height and weight were used to determine the body mass index of the participants (BMI). Additionally, waist and hip circumferences (WC & HC) were assessed via the usage of a measuring tape and measured from the central point between the lower rib margin and the iliac crest and at the maximal circumference over the buttocks. The measurement was performed when the participants were in a standing position. The waist-hip ratio (WHR) was determined as the ratio of WC to HC. The systolic and diastolic blood pressure were measured from the right arm of the participants while they were in a seated position using an automatic digital blood pressure measurement device (HEM-780, Omron, Kyoto, Japan). The body fat percentage (BF), total body water percentage (TBW Percentage), visceral fat (VF), bone mass, and muscle mass were measured using a digital body composition analyser (SC-330, Tanita, Japan) that applied the principles of bioelectrical impedance analysis (BIA).

#### 2.3.2. Nicotine Level Assessment

Nicotine levels were assessed using hair samples at the National Poison Centre (PRN) via gas chromatography-mass spectrometry (GCMS) [38]. The vortex posterior hair samples were cut off in close vicinity to the scalp, and only 10 cm (highest length) of each hair sample (gauged from the scalp end) was extracted. The hair sample was utilized to determine the level of nicotine in the body, a process that typically involves five important phases, namely, hair sampling, cleaning, digestion, extraction, and calculation. It is worth highlighting that this method was adopted in the study due to its superiority over the other methods (questionnaire-based), as it provides more accurate data for determining the precise level of nicotine in the body of a smoker.

#### 2.3.3. Physical Activity Level Estimation

The physical activity (PA) of the participants was assessed via the short version of the International Physical Activity Questionnaire (IPAQ). The IPAQ consists of seven constructs that measure various degrees of PA concerning intensity and duration at different times of the day. This instrument was administered to the participants to obtain their ratings based on the perceived daily PA. The scores were summed used to estimate both the durations and the frequencies of the performance of the various PPA smokers. The IPAQ is shown to be valid and reliable in estimating the PA levels among an adult population [39].

### 2.4. Informed Consent/Ethical Approval

Before the commencement of the current investigation, all the procedures and protocol were endorsed by the Research Ethics Committee (Human) of Universiti Sains Malaysia, and the study was conducted in compliance with the Helsinki Declaration for the experiment with human subjects. Moreover, informed consent was obtained from all the participants. All the experiments were carried out at the laboratory of the School of Medical Sciences, Universiti Sains Malaysia, Kubang Kerian, Kelantan.

### 2.5. Data Analysis

A variety of multivariate analyses and machine learning methods were used to answer the research questions previously raised and to achieve the objectives of the study.

#### Principal Component Analysis (PCA)

PCA is a mathematical method used primarily to identify the structure of a dataset from a group of observed variables [40]. PCA is often used as a data reduction technique to identify important variables for further analysis [41,42].

### 2.6. Application of PCA in the Study

In this study, the PCA was used to ascertain the AV associated with the smoking samples. This analysis is carried out to answer research question 1. The data retrieved via this procedure were outlined in the previous sections, i.e., age, weight, height, BMI, waist circumference, hip circumference, waist-hip ratio, fat percentage, muscle mass, total body water, bone mass, and nicotine were applied in analysing the data. All the variables were considered for PCA to identify the most important parameters for further analysis. Hence, a factor loading that is equal to or greater than 0.80, was considered important, while a variable that was smaller than the threshold was deemed less important [43]. It is worth noting that before commencing the full analysis in the current study, all the data acquired were standardized through z-score transformation, whereby the mean and the standard deviation of all variables were scaled to a z score. This method is deemed suitable for the removal of bias effects between variables [40,44].

#### Cluster Analysis (CA)

Clustering is one of the most common exploratory data processing techniques used to study the nature and patterns of datasets. Cluster analysis has been reported to be useful in identifying subsets or samples about certain observed variables [45,46].

### 2.7. The Application of k-Means Cluster Analysis in the Study

Cluster analysis was applied at this stage to partition the samples of smokers into groups based on similarities and differences in the PA scale scores. In the present investigation, a k-means clustering algorithm was used, and Euclidean distance was considered a distance metric for assigning the formation of the two clusters identified, i.e., partially physically active (PPA) and physically nonactive (PNA) smokers.

#### Discriminant Analysis (DA) and Logistic Regression (LR)

We employed DA in this study to determine the differences between the two clusters extracted via the clustering previously described, i.e., PPA and PNA smokers, based on their AV. At this stage, the anthropometric and health-related markers that are shown to be dominant via the PCA analysis were used. The anthropometric and health-related markers were considered the independent variables, whilst the smoking categories, i.e., PPA and PNA, were treated as the dependent variables. Three different modes of DA model, i.e., standard, backward, and forward stepwise, were developed to determine the best fit with the data.

Moreover, a logistic regression model (LR) was employed to further ascertain the efficacy of the discriminant models in classifying the two smoking groups. A five-fold cross-validation technique was used for developing the model [47]. The data obtained from each of the DA analyses were split into a ratio of 70:30 for training and test sets [46,48]. The Scikitlearn libraries were evoked for the development of the LR model via Spyder IDE. Other statistical analyses were implemented via XLSTAT2014 add-in software and Orange Canvas version 3.4.0 [49], Tržaška 25, SI-1000 Ljubljana, Slovenia, for Windows. All assumptions were deemed significant and drawn at an alpha level of *p* ≤ 0.05.

## 3. Results

Table 1 depicts the characteristics of the participants. The total number of participants and the maximum, minimum, mean age and standard deviation of the participants, as well as the smoking period, are tabulated. Some of the participants started smoking in late adolescence, i.e., at 20 years of age. The overall mean age of the participants was 37 ± 9.4 years, whilst the average duration of the participants’ smoking cessation histories was 16.9 ± 7.7 years. The cessation smoking history of the participants is also displayed. We observed that a total of 57 participants had attempted cessation, while 47 never attempted and 3 did not respond. It is worth highlighting that all participants were Malays recruited from different states in Malaysia. Many of the participants were urban dwellers, and the majority were from middle- to high-income families.

### 3.1. Identifying the Most Dominant AV Characteristics of Healthy Male Smokers

**Figure 1:**
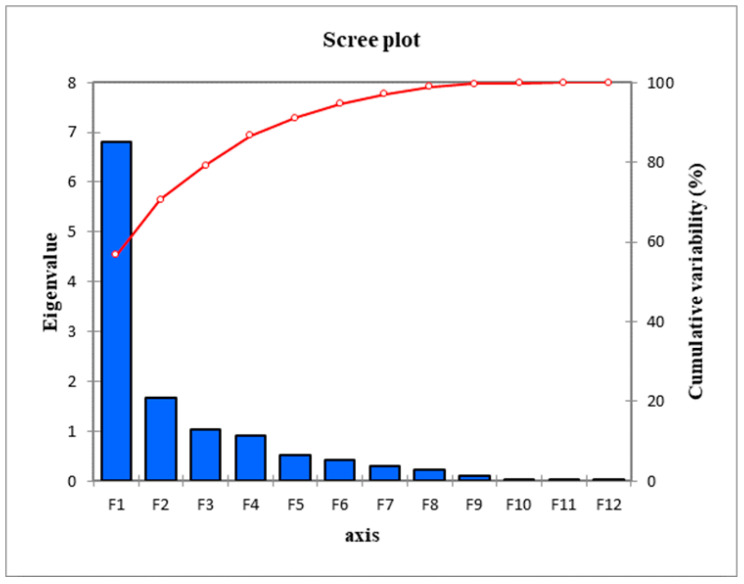
The eigenvalues from the initial PCA.

Figure 1 shows the scree plot of the eigenvalues for the PCA analysis. It could be observed from the figure that the PCA illustrated a total of three anthropometric components that are highly attributed to smoking behaviour. For each of these three components, some specific physical attributes are identified and considered the most affected due to their relatively higher Eigenvalues (greater than 1). These identified AV and health components were retained and subsequently used as inputs parameters for further analysis, i.e., varimax rotation.

Table 2 presents the PCA results after the varimax rotation, where some relevant anthropometric markers are revealed. These AV are identified due to satisfying the pre-set factor-loading threshold, i.e., greater or equal to 0.80. Likewise, it was observed that a total number of 7 AV, out of the 12 initially examined, were identified in all three components as more pronounced in smokers.

### 3.2. Identification of Smokers’ PA Groups and the Most Dominant AV Differentiating the Groups

**Figure 2:**
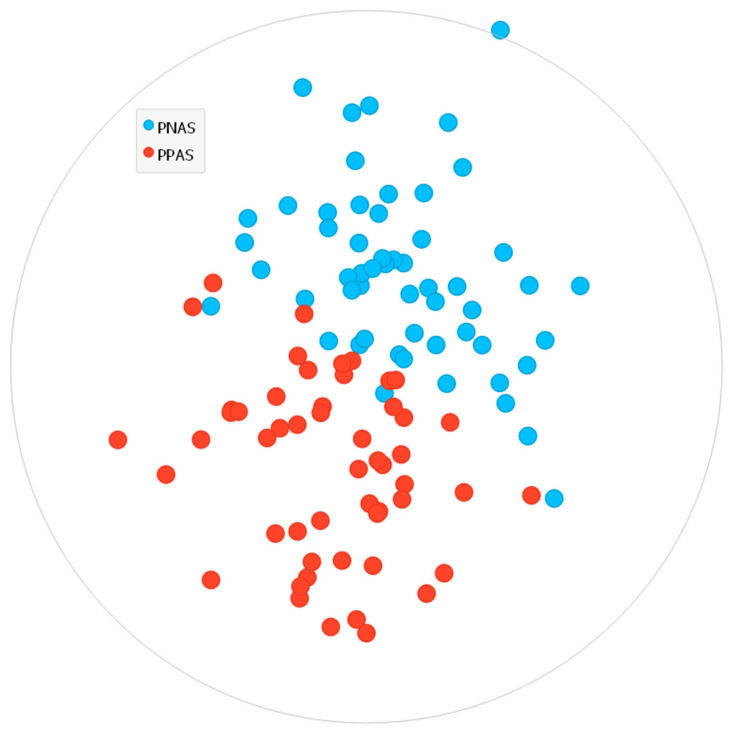
The smoking categories classified based on the PA levels via k-means algorithm.

Figure 2 displays the classes identified through the k-means analysis with respect to the PA levels of the smokers. It could be seen from the figure that a clear partition was established between the PPA and PNA smokers.

Table 3 indicates the differences in the measured variables between the smoking categories. It could be observed from the table that the PNA smokers possessed higher means of all anthropometric and health markers. However, in the physical activity levels from the IPAQ measurement, the PPA smokers recorded higher mean scores, which shows that the PPAS group spent more time engaging in physical activity compared with PNAS.

### 3.3. Examining the Effectiveness of the DA and Logistic Regression Models in Discriminating and Classifying the PPA and PNA Smokers Based on the AV Characteristics

To examine the efficacy of the DA and LR models in discriminating as well as classifying the PPAS and PNAS, we excluded the weight and height variables in the process of developing the model as more often than not, these variables were shown to be highly correlated [50].

Table 4 details the classification accuracies, discriminating the anthropometric variables as well as the confusion matrices of the DA models. It could be observed from the table that a total of 94.39 per cent classification accuracy was obtained from the standard model of the DA, with 1 misclassification in the PNAS and 5 misclassifications attributed to the PPAS. From the standard mode analysis of the DA, four AV consisting of BMI, waist circumference, fat per cent, and hip circumference significantly distinguished the two groups. Conversely, the backward and forward stepwise models demonstrated a total classification accuracy of 95.33 per cent, with 4 misclassifications in the PPAS cluster and 1 misclassification in PNAS. A total of three AV were observed as significantly differentiating the two clusters in the backward mode of the DA, namely, waist, fat percentage, and hip, whilst only two variables (fat percentage and hip circumference) were found to discriminate said clusters in the forward stepwise mode, with a total classification accuracy of 94.39 per cent and 4 misclassifications from the PPA smokers as well as 2 from PNAS. To determine the best model, the AV for each DA model was used to develop the LR model as shown in Table 4.

**Figure 3:**
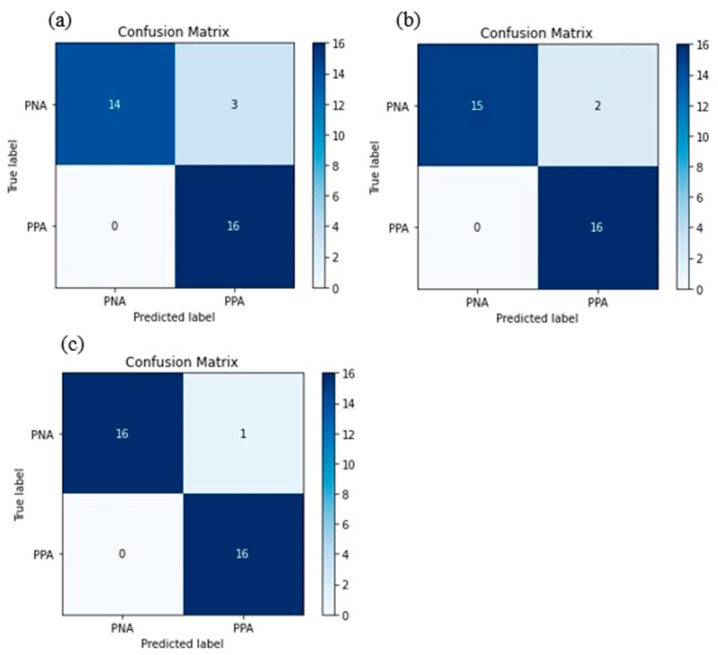
The confusion matrix for the LR models. (**a**) Standard DA feed-forward LR. (**b**) Backward DA feed-forward LR. (**c**) Forward stepwise DA feed-forward LR.

Table 5 reveals the parameters for the LR model developed. The classification accuracy, precision, recall, and F1 score for each model in both training and test are shown. It could be observed that the standard DA feed-forward LR exhibited CA of 1 and 0.91 for training and test respectively. The backward DA feed-forward LR revealed CA values of 1 and 0.94%, while the forward stepwise DA feed-forward LR demonstrated CA values of 0.96 and 0.97 for the training and the test sets, respectively. Meanwhile, Figure 3 displays the confusion matrix for the model. It could be observed from the confusion matrix that the standard and backward DA feed-forward LR demonstrated 3 and 2 misclassifications respectively, while the forward stepwise DA feed-forward LR showed only 1 misclassification. Therefore, it is evident that the forward stepwise DA feed-forward LR is the best model as it exhibits no overfitting behaviour, in contrast with the other two models.

The variables’ contributions to the efficacy of the LR models developed are tabulated in Table 6. The parameters for the models’ goodness of fit, which constitute values for the parameter estimate (value), beta, standard error, chi-square, and the corresponding *p* values, are shown. It could be observed from the table that fat percent and hip circumference were the highest contributors toward the prediction of group membership (PNAS or PPAS) in all three models developed. Hip circumference was observed to be a greater contributor to the model, with beta values of −3.6, −3.4, and −3.6, while fat percent had beta values of −3.1, −3.2, and −3.2 for each model, respectively. It is also evident that the best model (model c) has a comparatively lower SE as well as higher chi-square values for each variable, further demonstrating the importance of hip circumference and fat percentage in the model’s accuracy.

## 4. Discussion

What are the most dominant AV characteristics of healthy male smokers? The findings of the current investigation from the initial objective via PCA (Table 2) demonstrated that the most dominant AV characteristics of healthy male smokers could be identified in three principal components (PCs). The first PCs itemized weight, body mass index (BMI), waist and hip circumference, as well as per cent body fat. The second PCs projected body height, whilst the third PCs revealed nicotine level. These three principal components demonstrated that the smokers were characterized by higher body mass, high-fat accumulation, and a greater level of nicotine. This finding is concordant with the results reported from previous studies where the smoking samples were found to be heavier and with a considerable accumulation of body fat, as opposed to their non-smoking counterparts [51,52]. Moreover, a previous study demonstrated that the body fat of smokers is likely to be distributed mainly across the abdomen in a somewhat central or an apple-shaped pattern, which brings about adverse consequences for health [53]. It is worth noting that most of the effects of tobacco on body weight are regulated by nicotine, which induces the consumption of calories that could interfere with the weight gain processes of smokers [54].

What are the smokers’ PA levels and the most dominant AV that differentiate the PA groups? The characteristics and discriminating features of PNAS and PPAS are shown in Table 3 and Table 4, respectively. It was observed that weight, height, BMI, and waist and hip circumference as well as body fat per cent are higher in the PNAS compared with the PPAS. Previous investigation has established that an average smoker typically weighed less than similar aged non-smoker [17]. However, the current finding revealed that smokers who are physically inactive tend to be heavier compared with partially physically active smokers. It is plausible that physical inactivity’s effect on smoking is mainly attributed to weight gain, obesity, and high fat accumulation, which could pose a high risk of cardiovascular disease [55]. The persistent ingestion of tobacco has been reported to negatively influence metabolism, and smokers tend to consume 350 to 575 more calories per day as opposed to non-smokers [56]. Moreover, studies have demonstrated that smokers who live a sedentary lifestyle have lower physical performance and lack of endurance, characterized by shortness of breath, all of which could lead to the deterioration of overall health [57,58]. Therefore, it is important to note that lack of PA coupled with smoking is a harbinger of many health complications such as cardiovascular diseases and cancer that may affect the general well-being and, indeed, mortality, of an individual [34,59]. However, the adverse effects of smoking on overall health have been shown to be mitigable or reversable when an individual quits smoking. Thus, highlighting smoking cessation should remain a public health goal.

How effective are the DA and logistic regression models in discriminating and classifying the PPA and PNA smokers based on their AV characteristics? It is demonstrated from the best model of the current investigation, i.e., the forward stepwise DA feed-forward LR, that the major discriminating AV among the PA groups are the fat percentage and the hip circumference, as shown in Table 5. Previous studies documented that an increase in smoking coupled with a lack of PA resulted in fatness and larger waist or hip circumference after adjusting for BMI [55,60]. Regular PA has been shown to protect smokers from some adverse effects of smoking. Essentially, PA could help to prevent excessive weight gain and inflammation as well as muscle, loss [61,62]. It is also evident that the best model has a comparatively lower SE, as well as higher chi-square values for hip circumference and fat percent, further demonstrating the importance of hip circumference and fat percentage in the model accuracy, as reflected in Table 6. These variables, i.e., hip circumference and fat percent are found to be the highest contributors to the overall model accuracy in addition to being indicators for the prediction of PA group membership.

## 5. Conclusions

These major findings from our study revealed that partially physically active smokers are characterized by relatively desirable anthropometric variables, in contrast with physically non-active smokers. It is evident from the current findings that certain anthropometric variables are vital for maintaining good health and that physical activity is essential for maintaining a healthy body in a sample of smokers. Furthermore, physically non-active smokers are more inclined to develop cardiovascular problems, coupled with nicotine dependence, when compared with partially physically active. Finally, it is worth highlighting that the application of multivariate analysis and machine learning may be useful in studying the underlying associations between the investigated variables.

## 6. Practical Application and Future Direction

As a recommendation to diminish the prevalence of smoking among individuals, governments could decrease smoking pervasiveness through the increase of smoking expense, by methods of deploying tax increases, initiating constant social advertising efforts through which health educators could regularly encourage smokers to stop, and offering both pharmacological and humanitarian support for stopping smoking. The promotion of exercise as a daily activity could be an important mechanism through which the effects of smoking on health could be averted or controlled. Governments should embark on the creation of modern leisure and PA facilities in parks, streets, marketplaces, and institutions, as well as corporate buildings, to motivate and promote active lifestyles amongst people. From the public health and clinic perspectives, exercise as well as a variety of PA could be introduced as prescriptions as part of the cessation mechanism since exercise could assist in reducing cravings, managing other withdrawal symptoms, and diminishing stress.

## 7. Limitations of the Study

The current study is subject to certain limitations. For instance, the dietary intake of the participants was not assessed, which could provide more precise characteristics of the participants with respect to their PA levels. The findings of the current investigation also could not be generalized to female smokers. The lack of the use of an actual PA test may have hindered the accurate estimation of the PA levels among the participants since over- or under-reporting of the PA levels by the participants could not be completely ruled out. Moreover, the use of physical assessment batteries, as well as other motor-related functional tests, could be explored to determine the physical fitness levels of smokers in future studies.

## Figures and Tables

**Table 1:** The Study Participants’ Characteristics.

Variables	N	Minimum	Maximum	Mean (SD)
Participant Age (years)	107	20	55	37 (9.4)
Smoking Period (years)	107	1	35	16.9(7.7)
Smoking Cessation History	N	Percent		
Yes	57	53.3		
Never	47	43.9		
No response	3	2.8		
Total	107	100		

**Table 2:** Principal component analysis results after rotation.

Variables	PC1	PC2	PC3
Age (years)	0.254	−0.319	0.583
Weight (kg)	0.842 *	0.504	0.047
Height (cm)	0.042	0.922 *	−0.046
BMI (kg/m^2^)	0.945 *	0.123	0.078
Waist (cm)	0.835 *	0.163	0.091
Hip (cm)	0.811 *	0.417	0.006
WHR	0.755	−0.151	0.255
Fat Percentage (%)	0.825 *	0.146	0.015
Muscle Mass (kg)	0.674	0.718	0.019
TBW (%)	−0.796	−0.142	0.092
Bone Mass (kg)	0.653	0.724	0.028
Nicotine	−0.092	0.169	0.843 *
Eigenvalue	6.798	1.671	1.025
Variability (%)	56.652	13.926	8.543
Cumulative %	56.652	70.577	79.12

Note: * = most dominant variables, WHR = Waist hip ratio, PC = principal component. TBW = Total body water.

**Table 3:** The differences in the anthropometric and health variables between the physically non-active and the partially physically active smokers.

	Physical Activity Groups		
Variables	Mean (SD)		
	PNAS (*n* = 55)	PPAS (*n* = 52)	*p*-Values
Weight (kg)	79.6 (8.0)	59.9 (7.1)	0.001
Height (cm)	170.1 (6.4)	165.2 (6.0)	0.001
BMI (kg/m^2^)	27.5 (2.5)	22.0 (2.6)	0.001
Waist (cm)	92.2 (5.6)	74.9 (9.9)	0.001
Fat Percentage (%)	25.9 (5.1)	17.4 (4.4)	0.001
Nicotine	7.4 (9.9)	6.2 (6.4)	0.001
Hip (cm)	102.5 (4.1)	90.5 (4.7)	0.451
IPAQ (MET-minutes/week)	4095.3 (2458.1)	4429.5 (2828.0)	0.515

Note: PNAS = physically nonactive smokers, PPAS = partially physically active smokers.

**Table 4:** The discriminant analysis model of the smoking groups.

Assigned Classes	% Correct	Classification Matrix Assigned by DA	
		PPAS	PNAS
Standard Mode (BMI, Waist, Fat%, Hip)			
PPAS	90.38%	47	1
PNAS	98.18%	5	54
Total	94.39%	52	55
Backward Mode (Waist, Fat Percentage, Hip)			
PPAS	92.31%	48	1
PNAS	98.18%	4	54
Total	95.33%	52	55
Forward Stepwise (Fat Percentage, Hip)			
PPAS	92.31%	48	2
PNAS	96.36%	4	53
Total	94.39%	52	55

**Table 5:** The Logistic Regression Model for the PA Smoker Groups.

			Model Parameters			
Model Types	Model Evaluation	PA Groups	CA	Precision	Recall	F1 Score
Standard DA Feed-forward LR	Training	PNA	1.0	1.0	1.0	1.0
		PPA		1.0	1.0	1.0
	Test	PNA	0.91			
		PPA		1.0	0.82	0.9
Backward DA Feed-forward LR	Training	PNA	1.0	0.84	1.0	0.91
		PPA		1.0	1.0	1.0
	Test	PNA	0.94	1.0	0.88	0.94
		PPA		0.89	1	0.94
Forward stepwise DA Feed-forward LR	Training	PNA	0.96	0.95	0.97	96
		PPA		0.97	0.94	0.96
	Test	PNA	0.97	1.0	0.94	0.97
		PPA		0.94	1.0	0.97

Note: PA = Physical activity, CA = Classification accuracy.

**Table 6:** The contributions of the variables to the LR model’s efficacy.

Models and Variables	Model Parameters				
Model (a)	Value	Beta	SE	Chi-Square	*p*-Values
Intercept	104.6		35.5	8.7	
BMI	0	0	0	0	
Waist	0	0	0	0	
Fat Percent	−0.9	−3.1	0.4	5.6	0.018
Hip	−0.9	−3.6	0.3	10.2	0.001
Model (b)					
Intercept	102.1		40.9	6.2	
Waist	0	0	0	0	
Fat Percent	−0.9	−3.2	0.4	5.8	0.016
Hip	−0.9	−3.4	0.3	9.7	0.002
Model (c)					
Intercept	104		31.8	10.7	
Fat Percent	−0.8	−3.2	0.4	5.7	0.017
Hip	−0.9	−3.6	0.3	10.2	0.002

Note: SE = Standard Error, model (a) = Standard DA feed-forward LR, model. (b) = Backward DA feed-forward LR. model (c) = Forward stepwise DA feed-forward LR.

## Data Availability

The data used is available within the manuscript.
